# Long‐term prognosis in kidney transplant recipients with human T‐cell leukemia virus type 1 infection

**DOI:** 10.1111/tid.13314

**Published:** 2020-05-21

**Authors:** Norihiko Goto, Kazuharu Uchida, Toshihide Tomosugi, Kenta Futamura, Manabu Okada, Takahisa Hiramitsu, Shunji Narumi, Yoshihiko Watarai

**Affiliations:** ^1^ Department of Transplant Nephrology Nagoya Daini Red Cross Hospital Nagoya Japan; ^2^ Department of Transplant Surgery Masuko Memorial Hospital Nagoya Japan; ^3^ Department of Transplant Surgery Nagoya Daini Red Cross Hospital Nagoya Japan

**Keywords:** HTLV‐1, kidney transplantation, outcome

To the Editor

Human T‐lymphotropic virus (HTLV‐I), identified as the first human retrovirus in 1980, can cause fatal diseases which are HTLV‐I‐associated myelopathy/ tropical spastic paraparesis (HAM/TSP) and adult T‐cell leukemia‐lymphoma (ATL).^1^, ^2^ It could be transmitted from transplanted organs,^3^ as well as three main modes of transmission which are mother to child by breast feeding, blood transfusion, and sexual intercourse. Immunosuppressive agents can accelerate reactivation of HTLV‐1; therefore, kidney transplant recipients (KTR) who are HTLV‐1 positive might be at risk for the onset of HAM–TSP and ATL. The risk of HAM–TSP and ATL after kidney transplantation (KT) was revealed last year.^3^ However, 4.5 years as a follow‐up period in this paper is not long enough for comparing the onset of HAM‐TSP and ATL to that of general population. The cumulative lifetime risk of ATL in a HTLV‐I infected patient is between 2 and 5 percent. Twenty to thirty years might be required from viral infection to the onset of ATL in general population.^4^

Since 1972, we performed 46 KT for 41 candidates with HTLV‐1. Long‐term outcome is shown in Table 1. Median follow‐up period included restarting dialysis was 11 years and half. One (2%) developed HAM–TSP from a HTLV‐1–negative living donor at 8 years after KT. He died with functioning graft 3.5 years after onset of HAM–TSP because of sepsis caused from streptococcus pneumonia. Another (2%) developed ATL from a HTLV‐1–negative living donor at 22 years after KT. She has being under treatment.

**Table 1:** Characteristics and outcomes of KTR with HTLV‐1

Characteristic or Outcome	Overall (41 KTR, 46 KT)	Donor pos, recipient pos (2 KTR)	Donor neg, recipient pos (39 KTR, 44 KT)
1st/2nd KT	41/5	2/0	39/5
Median post‐KT period (included restarting dialysis) (range)—mo	140 (0‐556)	104 (22‐186)	140 (0‐556)
Median post‐KT period using immunosuppressive agents (range)—mo	111 (0‐453)	104 (22‐186)	111 (0‐453)
Living related KT—no. (%)	32 (70)	2 (100)	30 (68)
Median age at 1st KT (range)—yr	40 (18‐70)	55 (40‐70)	39 (18‐69)
Male sex—no. (%)	30 (73)	2 (100)	28 (72)
HAM–TSP—no. (%)	1 (2)	0 (0)	1 (3)
Time from KT to HAM–TSP (range)—mo	99	NA	99
ATLL—no. (%)	1 (2)	0 (0)	1 (3)
Time from KT to ATLL—mo	244	NA	244
DWFG — no. (%)	13 (32)	0 (0)	13 (30)
Median time from KT to DWFG (range)—mo	120 (0‐423)	NA	120 (0‐423)
Graft loss excluded DWGF—no. (%)	14 (34)	0 (0)	14 (32)
Median time from KT to graft loss excluded DWFG (range)—mo	15 (0‐370)	NA	15 (0‐370)
Death after graft loss—no. (%)	3 (7)	0 (0)	3 (7)
Median time from KT to death after graft loss (range)—mo	140 (1‐184)	NA	140 (1‐184)

Abbreviations: ATLL, adult T‐cell leukemia‐lymphoma; DWFG, death with functioning graft; HAM‐TSP, HTLV‐I‐associated myelopathy/ tropical spastic paraparesis; KT, kidney transplantation; KTR, kidney transplant recipients; mo, month; neg, negative; pos, positive; pts, patients; yr, year.

Although HTLV‐1–negative recipients who received KT from HTLV‐1–positive living donors have not been accepted in our institution, KT for candidates with infected HTLV‐1 from HTLV‐1–positive living donors has not being contraindicated in Japan.

HTLV‐I displays little genomic variability in the same geographic endemic area, such as southwestern part of Japan. Therefore, there is little possibility of superinfection.

None of candidates with HTLV‐1 from HTLV‐1–positive living donors have experienced HAM‐TSP or ATL during the follow‐ups.

This data is small and from single center; however, the long‐term outcome is sufficient to be accepted. KT may be the optimal renal replacement therapy even for HTLV‐1 infected candidates.

## AUTHOR CONTRIBUTIONS

NG wrote the manuscript. NG, KU, TT, KF, MO, TH, SN, and YW collected the samples and data. NG contributed study design and analysis. All authors participated in revising it critically for important intellectual content and approved the final version for publication.
